# Effects of essential oil of *Origanum onites* and its major component carvacrol on the expression of toxicity pathway genes in HepG2 cells

**DOI:** 10.1186/s12906-024-04571-6

**Published:** 2024-07-11

**Authors:** Özlem Tomsuk, Victor Kuete, Hülya Sivas, Mine Kürkçüoğlu

**Affiliations:** 1 Cellular Therapy and Stem Cell Production Application and Research Centre (ESTEM), Eskisehir Osmangazi University, Eskisehir, 26480 Turkey; 2 Graduate School of Natural and Applied Sciences, Biotechnology and Biosafety Department, Eskişehir Osmangazi University, Eskişehir, Turkey; 3 Faculty of Sciences, Department of Biology, Anadolu University, Eskişehir Technical University, Eskişehir, Turkey; 4 Department of Biochemistry, Faculty of Science, University of Dschang, P.O. Box 1499, Bafoussam, Cameroon; 5 Department of Pharmacognosy, Faculty of Pharmacy, Anadolu University, Eskişehir, Turkey

**Keywords:** Carvacrol, Essential oil, Hepatotoxicity, HepG2, Origanum onites, RT^2^-PCR array

## Abstract

**Background:**

*Origanum* species have been used in various commercial constructions as a remedy against burns and wounds, agriculture, alcoholic drinks, fragrance, and flavoring substances of food products. The essential oil of *Origanum onites* L. (EOOO) and its component carvacrol (CV) possesses a wide range of biological activities including anti-cancer activity.

**Purpose:**

The purpose of this study was to investigate the growth inhibitory activity of the essential oil and its major component CV and then hepatotoxicity pathway-related genes in HepG2 cells.

**Methods:**

The effects of the EOOO and CV on cell growth and mRNA expressions of 84 hepatotoxicity pathway-related genes were investigated in HepG2, using trypan blue exclusion/ bromodeoxyuridine (BrdU) incorporation tests and real-time-polymerase chain reaction (RT-PCR) array, respectively.

**Results:**

The EOOO and CV inhibited cell growth with IC_50_ values of 0.08 µg/mL and 45 µg/mL, respectively, after 24 h. Real-time, reverse-transcription-polymerase chain reaction (RT^2^-PCR) array analysis revealed that expressions of 32 genes out of 84 were changed at least 2-fold or more in the EOOO-treated cells. Among them, expression levels of 17 genes were elevated, while expression levels of 15 genes were diminished. Furthermore, after exposure of cells to 45 µg/mL of CV, the expression of 8 genes was increased while the other 8 genes were decreased. Both the EOOO and carvacrol affected the expression of 48 genes of HepG2 cells which are involved in the hepatotoxicity pathway, indicating their hepatoprotective and possible anti-hepatocarcinogenic effects.

**Conclusion:**

The present study demonstrates that the essential oil of *Origanum onites* and carvacrol can be used in various applications such as anticancer or herbal drugs, since its non-hepatotoxicity.

## Background

Natural plant-derived compounds have received increased attention from scientists for their potential in several medicinal applications, including cancer treatment, as recently reported by several studies [1]. Extracts, essential oils, and compounds from *Origanum* species have been used in various traditional health systems or commercial constructions as remedies against microbial infections, burns, and wounds, in agriculture, and also as alcoholic drinks, fragrance, and flavoring substances for food products The anti-cancer, anti-proliferative, and apoptotic effects of the EOOO and several extracts from *Origanum* species were investigated on various cell types including leukemic cells, platelets, breast adenocarcinoma cells, human fibroblasts, murine melanoma cells, colon adenocarcinoma, and hepatocarcinoma cells [2, 3].

The present study focused on the essential oil of *Origanum onites* L. (Lamiaceae) and its constituent, 2-methyl-5-propan-2-ylphenol or carvacrol (CV). The EOOO has been shown to possess various biological properties such as anticancer [4–7], antioxidant [5, 8], antimicrobial and antiviral [9], and antiangiogenic [10] activities. CV is one of the predominant monoterpene phenols present mainly in many essential oils including thyme and oregano [11, 12]. CV has also been shown to possess numerous biological similar to the EOOO in addition to cytotoxic, genotoxic, anti-mutagenic, and antioxidant [11, 13–16]. Specifically, earlier reports introduced the inhibitory effects of CV on tumorigenesis in rats [17–19]. Moreover, growth inhibitory effects of CV have been indicated in numerous studies in human or mammalian cancer cells both in vitro and in vivo [14, 20–23].

Despite numerous reports on the biological activities of the EOOO and CV, there have been few investigations performed at the level of the affected gene expression profile. Lee et al. [24] suggested that carvacrol induces the expression of the type I collagen gene *via* the Phospholipase C gamma (PLC-γ) signaling pathway. Following the activation of the Activator Protein-1 (AP-1) promoter, c-Jun N-terminal kinases (JNK) and extracellular signal-regulated kinase 1/2 (ERK1/2) (p42/44 (mitogen-activated protein kinase (MAPK)) were phosphorylated, but phosphorylation p38 MAPK did not occur in human dermal fibroblasts. In another work, visceral adipogenesis was inhibited by CV, probably through the suppression of bone morphogenic protein, fibroblast growth factor 1- and galanin-mediated signaling, toll-like receptor 2 (TLR2)- and TLR4-mediated signaling [25]. Kianmehr et al. [26] reported the immunomodulatory effect of carvacrol due to increased interferon-gamma (IFN-γ) and Forkhead box protein P3 (FOXP3) but decreased interleukin (IL)-4, transforming growth factor-beta (TGF-β), and IL-17 genes expression. In human macrophage-like U937 cells, CV exerted its anti-inflammatory activity by suppressing lipopolysaccharide-induced cyclooxygenase-2 (COX-2), mRNA and protein expression, and regulating COX-2 expression through its agonistic effect on peroxisome proliferator-activated receptor gamma (PPAR-γ). Recently, an investigation utilizing genomic profiling revealed a transcriptional response to CV closely resembling that of calcium stress in *Saccharomyces cerevisiae*. Genes involved in alternate metabolic and energy pathways, as well as stress response, were prominently upregulated while genes mediating ribosome biogenesis and RNA metabolism were repressed [27]. Kim et al. demonstrated that the expression of 74 genes has changed in intestinal intraepithelial lymphocytes of CV-fed chickens examined by microarray analysis [28].

Hepatocellular carcinoma (HCC) is the most common type of liver cancer globally and the second most common cause of cancer mortality, currently lacking effective diagnosis and treatment options [29, 30]. For decades, multiple natural novel therapeutic agents have been evaluated for HCC [31–33]. On the other hand, some researchers have focused on herbal hepatotoxicity (liver damage/injury), which persists as a concern for certain herbal drugs or plants used in the treatment of liver cancers. Certain herbs have been suggested as the cause of hepatotoxicity such as *kava kava* and as a result, many herbals have been implicated in herb-induced liver injury [34–36]. Newly, it has become well known that some plants may be hepatotoxic due to major compounds/secondary metabolites [37–39].

In our previous work, we showed that EOOO and CV inhibited the growth of human hepatocarcinoma cells, HepG2 [40]. In the present study, our main aim was to investigate the growth inhibitory activity of the essential oil and its major component CV, and subsequently assess hepatotoxicity-related genes in HepG2 cells. The hepatotoxicity of EOOO and CV was evaluated at the level of gene expression involved in mechanisms related to hepatotoxicity.

## Methods

### The essential oil of *Origanum onites* and CV

**Fig. 1:**
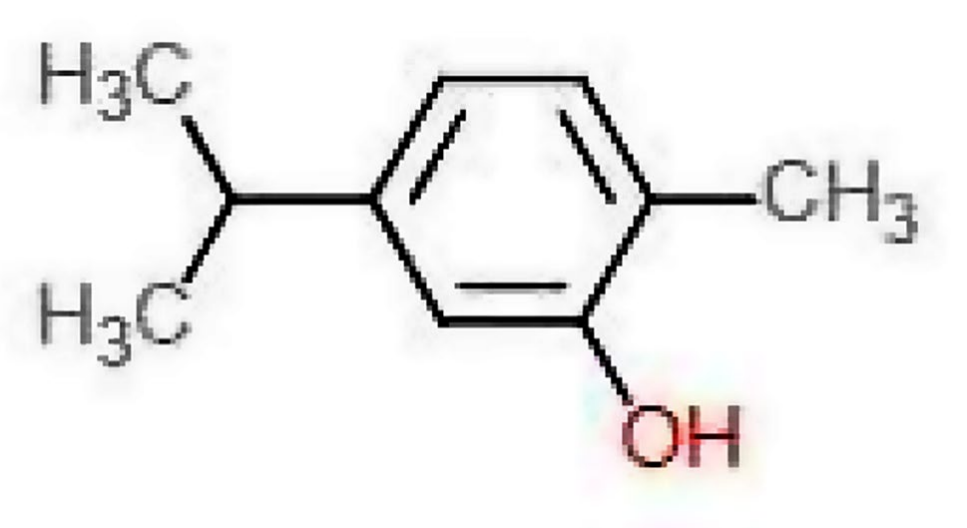
Chemical structure of CV

The plant material was acquired from Türer Inc. (İzmir, Turkey). This plant is not an endangered species, and no permission is required for its collection. The EO was extracted from the whole air-dried aerial parts of the *Origanum onites* L. (batch number: Wfo-000026069) by steam distillation for 3 h using Clevenger apparatus to produce the EO. The EOOO was further analyzed by Gas Chromatography (GC) and Gas Chromatography-Mass Spectrometry (GC-MS) using an Agilent GC-MSD system (Mass Selective Dedector-MSD). The composition of the EOOO has been analyzed before as given in Table 1. CV (2-methyl-5-propan-2-ylphenol) (Fig. 1) was purchased from Sigma-Aldrich (Taufkirchen, Germany). The stock solution was prepared in DMSO and diluted to the concentrations of the EOOO (0.02–0.1 µg/mL) and CV (7.5–120 µg/mL) freshly in the media for each experiment. The final concentration of DMSO in the cells was not more than 0.1% (v/v), and concentrations were prepared with the same amount of solvent in all doses.

**Table 1:** Major compounds of essential oil of *Origanum onites* L. analysed by GC/ GC-MS.

RRI	Main compounds	%	IM
**1188**	α-Terpinene	0.6	t_R_, MS
**1255**	γ-Terpinene	0.7	t_R_, MS
**1280**	*p*-Cymene	7.3	t_R_, MS
**1553**	Linalool	3.8	t_R_, MS
**1611**	Terpinen-4-ol	1.8	t_R_, MS
**1612**	β-Caryophyllene	0.7	t_R_, MS
**1706**	α-Terpineol	0.5	t_R_, MS
**1719**	Borneol	1.1	t_R_, MS
**1737**	β-Bisabolene	2.0	t_R_, MS
**2205**	Thymol	7.6	t_R_, MS
**2239**	Carvacrol	72.0	t_R_, MS

RRI: (Relative retention indices) calculated against n-alkanes,%: calculated from FID (Flame ionization detection) data; IM, identification method: tR, identification based on the retention times (tR) of genuine compounds on the HP Innowax column; MS, identified based on computer matching of the mass spectra with those of the Wiley and Mass Finder libraries and comparison with literature data

### GC-MS analysis and GC

The GC-MS analysis was carried out with an Agilent 5975 GC-MSD system (Agilent, USA; SEM Ltd., Istanbul, Turkey). Innowax FSC column (60 m x 0.25 mm, 0.25 μm film thickness) was used with helium as carrier gas (0.8 mL/min.). GC oven temperature was kept at 60 °C for 10 min and programmed to 220 °C at a rate of 4 °C/min and kept constant at 220 °C for 10 min and then programmed to 240 °C at a rate of 1 °C/min. The split ratio was adjusted to 40:1. The injector temperature was 250 °C. MS were taken at 70 eV. The mass range was from m/z 35 to 450. The GC analysis was carried out using an Agilent 6890 N GC system. To obtain the same elution order with GC/MS, a simultaneous injection was done by using the same column and an appropriate operational condition [41]. FID temperature was 300 °C.

### Cell culture

A human liver hepatocellular carcinoma cell line HepG2 purchased from DSMZ (Braunschweig, Germany) was cultured in DMEM (Dulbecco Modified Eagle Medium) (Sigma) supplemented with 10% FBS (fetal bovine serum) (PAA Lab. GmbH, Les Mureaux, France), penicillin/streptomycin at 100 units/ml and 2 mM L-glutamine as adherent monolayers. Cultures were incubated at 37^o^C under 5% CO_2_ / 95% air in a humidified atmosphere. Cells were passaged and harvested by 1% trypsin/EDTA (Sigma).

### Analysis of DNA synthesis by BrdU incorporation

DNA synthesis was monitored by measuring the incorporation of thymidine analogue 5-Bromo-2′-deoxyuridine (BrdU) in growing cancer cells using BrdU labeling. This method is based on the detection of BrdU incorporated into the genomic DNA of proliferating cells. The BrdU colorimetric kit (Cell Proliferation ELISA, BrdU Kit; Roche Molecular Biochemical, Germany) was used to determine the DNA synthesis by the method given by the manufacturer. Briefly, HepG2 cells were subcultured into 96-well tissue-culture microplates at a density of 1 × 10^4^/mL and incubated for 24 h. Then cells were incubated with or without a serial concentration of EOOO (0.02, 0.04, 0.05, 0.06, 0.07, 0.08, 0.09 and 0.1 µg/mL) and CV (7.5, 15, 30, 45, 60, 75, 90 and 105 µg/mL) and DMSO-treated cells for further 48 h. The cells were labelled by the addition of 10 µL BrdU solution (Roche Applied Science) for 2 h at 37^o^ C. Then cells were fixed and denatured by the addition of 200 µL of FixDenat for 30 min at room temperature. After removing the fixDenat solution, cells were treated with 100 µL of anti-BrdU-POD antibody solution (1:100) for 90 min at room temperature. Then the cells were washed three times with PBS and incubated with 100 µL of substrate solution for 15 min at room temperature. The absorbance of the samples was measured at 490 nm in an ELISA reader (ELX 808 IU, Biotek Instruments Inc., USA). After removing the labeling medium, the cells were fixed, and the DNA was denatured in one step by adding FixDenat. Three independent biological replicates, each performed in quadruplicate were performed. The values of the blank wells were subtracted from each well of treated and control cells.

### Trypan blue exclusion test

HepG2 cell viability was determined by the trypan blue (TB) exclusion assay. In our previous study, we demonstrated the cytotoxic effects of EOOO and CV through cell viability assessments utilizing WST-1 and Neutral Red assays on HepG2 cells [40]. The IC_50_ values (IC_50_ of EOOO about 0.09 µg/mL and CV about 75 µg/mL in HepG2 cells) and a sub-dose were determined based on the results of the cell viability assays, which were further confirmed using the trypan blue exclusion test. The dye exclusion test was used to determine the number of viable cells by counting the unstained cells. HepG2 cells (3 × 10^5^ cells/well) were grown in 6-well culture plates for 24 h and then exposed to different concentrations of EOOO (0.08 µg/mL and 0.09 µg/mL) and CV (45 µg/mL and 75 µg/mL) for an additional 24, 48, 72 and 96 h. Floating and adhering cells were collected and stained with 0.04% trypan blue at room temperature before they were examined under an inverted microscope. The proliferation rate was calculated based on the daily number of viable cells and presented on the graph. At least three separate experiments were performed in quadruplicate.

### Analysis of gene expression by real time PCR (RT^2^-PCR) array profiler

Gene expression profiles were obtained from HepG2 cells treated with EOOO and CV using the Human Hepatotoxicity RT² Profiler PCR Array (cat no. PAHS-093Z, 84 genes covered, SABiosciences, Qiagen, (Hilden, Germany). This Array profiles the expression of 84 key genes implicated as potential biomarkers of liver toxicity which are related to hepatotoxicity, involved in pathways of cholestasis, steatosis, nongenotoxic hepatocarcinogenicity, phospholipidosis, and necrosis (Table 1). The total RNA was extracted from 2.5 × 10^6^ cells treated with 0.08 µg/mL EOOO and 45 µg/mL CV or only 0.001% DMSO as a solvent control for 24 h using a RNeasy Mini Kit (Qiagen) and RNA samples were treated with RNase-free DNase (Qiagen) according to the manufacturer’s protocol. The cell lysate was homogenized by using QIAshredder. The final concentration of total RNA was determined using the Nanodrop DA-1000 Spectrophotometer. Only samples at a ratio between 2.0 and 2.1 were used for cDNA synthesis with an absorbance of 260/280 nm. Reverse transcription of 2 µg of total RNA into cDNA was performed using the RT² First Strand Kit (SABiosciences, Qiagen). The RT²-PCR was performed on Stratagene Mx3005P QPCR System (Thermo Fischer Scientific) using the Human Hepatotoxicity RT² Profiler PCR Array plate and RT² SYBR Green Master Mix (SABiosciences, Qiagen) according to the manufacturer’s protocol. Thermal profile was set as Segment 1 (1 cycle): 10 min at 95^o^C; Segment 2 (40 cycles): 15 s at 95^o^C, 1 min at 60^o^C; Segment 3 (1 cycle): 1 min at 95^o^C, 30 s at 55^o^C, 30 s at 95^o^C. The mRNA expression levels in the EOOO and CV-treated cells were compared to solvent-treated control cells and fold changes of gene expression were analyzed by using PCR array online data analysis (https://www.qiagen.com/tr/shop/genes-and-pathways/data-analysis-center-overview-page). The data analysis was normalized against the housekeeping genes by calculating the 2-^Δ^Ct in the plate. Genomic DNA contamination controls, RT negative, and positive controls were also tested for each array plate. Experiments were repeated as at least three independent trials.

### Statistics

For most experiments, mean values were compared with controls by Dunnett’s in One-way ANOVA, SPSS to evaluate statistical differences. The p values are calculated based on a Student’s t-test of the replicate 2^ (- Delta CT) values for each gene in the control group and treatment groups for PCR array analysis.

## Results

### Identification of major compounds of EOOO

The components of essential oils were identified by comparison of their mass spectra with those in the Baser Library of Essential Oil Constituents, Adams Library [42], Mass Finder Library, Wiley GC/MS Library [43] and confirmed by comparison of their retention indices. These identifications were accomplished by comparison of retention times with authentic samples or by comparison of their relative retention index (RRI) to a series of n-alkanes. Alkanes were used as reference points in the calculation of RRI [44]. Relative percentage amounts of the separated compounds were calculated from FID chromatograms. The results of the analysis are shown in Table 1. As shown in Table 1, the main component of EOOO was CV at 72%. Other components were thymol 7.6%, *p*-cymene 7.3%, linalool 3.8%, β-bisabolene 2.0%, terpinen-4-ol 1.8%, borneol 1.1%, β-caryophyllene 0.7%, γ-terpinene 0.7%, α-terpinene 0.6% and α-terpineol 0.5%.

### The EOOO and its component CV inhibit DNA synthesis in HepG2 cells

**Fig. 2:**
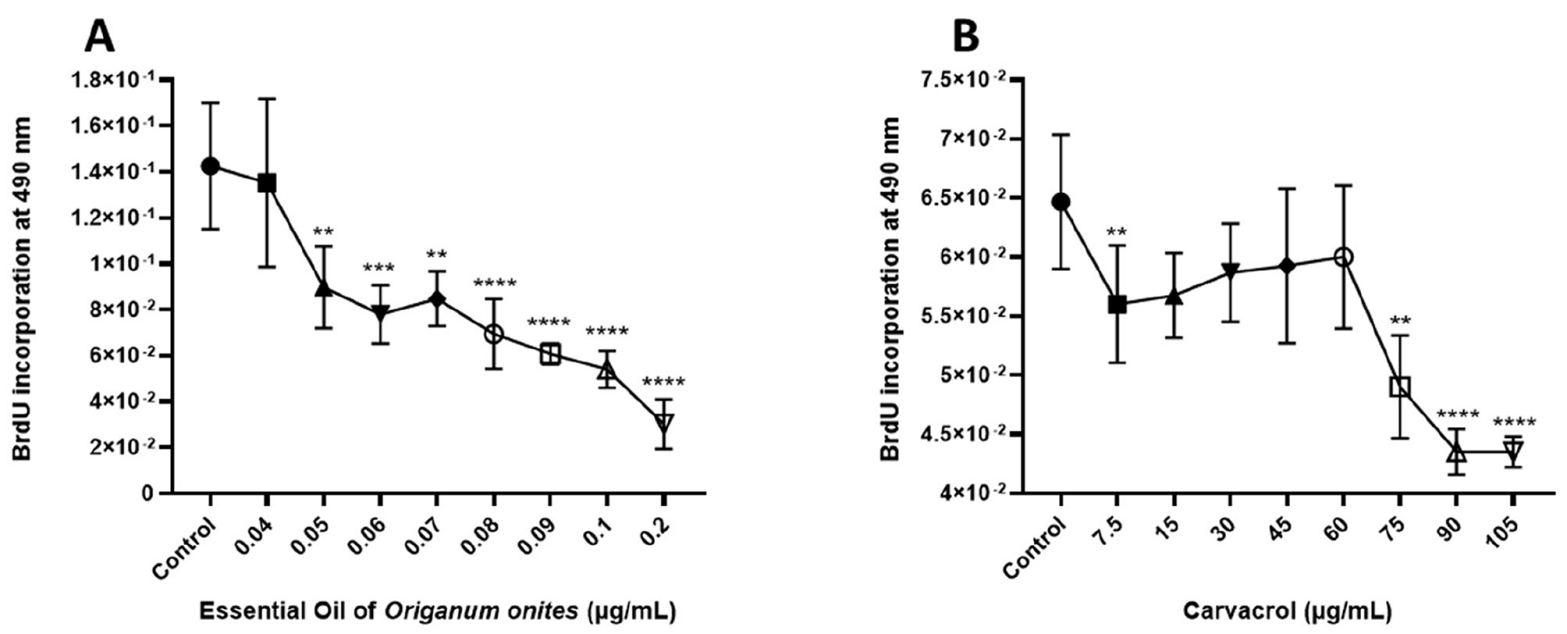
Inhibition effects of DNA synthesis from the EOOO. (0.02–0.2 µg/mL) (**A**) and CV (7.5–105 µg/mL) (**B**) on the growth of HepG2 cells after 48 h. The EOOO and CV induced inhibition of DNA syntheses were in a concentration-dependent manner by the method BrdU incorporation assay. All values are expressed as mean ± SD at least three separate experiments performed in quadruplicate. Differences were considered significant compared to the control group from *p ≤ 0.05, ** *p* ≤ 0.01, *** *p* ≤ 0.001, **** *p* ≤ 0.0001

The level of DNA synthesis was evaluated following treatment of HepG2 cells with the EOOO and CV for 48 h, using BrdU incorporation assay. The results were expressed in terms of the relative absorbance of EOOO and CV in comparison to control cells. As shown in Fig. 2, a concentration-dependent decrease in DNA synthesis of HepG2 cells was observed with increasing concentrations of both EOOO and CV (*p* < 0.05). DNA synthesis was significantly declined in the cells by the concentration of 0.06 µg/mL of EOOO as compared to solvent-treated cells as a control. Inhibition of DNA synthesis by the EOOO was determined as around 50% percent at the concentration of 0.08 µg/mL (Fig. 2A). CV also inhibited the DNA synthesis in the cells in a similar way. DNA synthesis was significantly declined in the cells by the concentration of 60 µg/mL of CV concentration-dependent manner as compared to solvent-treated cells as a control (Fig. 2B).

### The EOOO and CV suppress cell proliferation in HepG2 cells

**Fig. 3:**
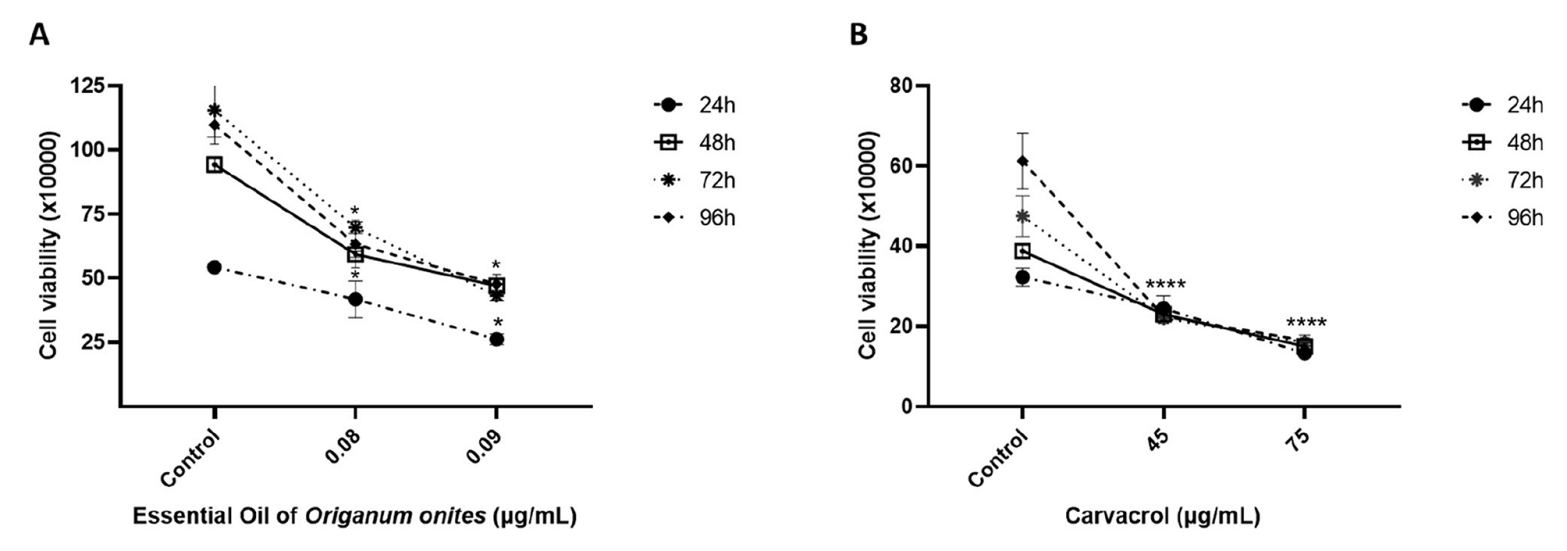
Effects of the EOOO (**A**) (0.08 and 0.09 µg/mL) and (**B**) CV (45 and 75 µg/mL) on viability of HepG2 cells incubated for 24, 48, 72 and 96 h. Each value is the mean ± S.D. of three separate experiments performed in quadruplicate. Differences were considered significant compared to the control group from *p ≤ 0.05, ** *p* ≤ 0.01, *** *p* ≤ 0.001, **** *p* ≤ 0.0001

The viability of cells was determined by Trypan Blue (TB) exclusion assay after incubation in the presence of the EOOO and CV for periods. Concentrations of EOOO and CV were chosen based on the results of our previous WST-1 and neutral red assay [40]. According to the cytotoxicity test results, IC50 values were approximately determined as 0.09 µg/mL for EOOO and 75 µg/mL for CV. Therefore, for verification of the dose to be applied in microarray studies, the trypan blue test was also performed by selecting the IC50 value and a sub-dose. Cells were exposed to 0.08 and 0.09 µg/mL concentrations of EOOO and 45 and 75 µg/mL concentrations of CV for 24, 48, 72, and 96 h, then stained with TB. As shown in Fig. 3, the number of viable cells was diminished in a time and concentration-dependent manner similar to the results obtained by BrdU assay. The number of viable cells dropped to 65% at the concentration of 0.08 µg/mL EOOO and to 51% at the concentration of 0.09 µg/mL EOOO after 48 h (*p* < 0.05) (Fig. 3A). Also, the number of viable cells significantly was decreased to 39% at the concentration of 0.09 µg/mL EOOO after 72 h. The IC_50_ value of the EOOO was determined to be 0.09 µg/mL. at 24 and 48 h.

CV was also cytotoxic for HepG2 cells, similar to the effect of EOOO (Fig. 3B). The number of cells was significantly diminished at the concentrations of 45 and 75 µg/mL CV when compared to solvent-treated control cells. The viability of cells was 64% after 24 h and 45% after 48 h treatment with 75 µg/mL CV, and 54% after 72 h and 50% after 96 h treatment with 45 µg/mL. Therefore, IC_50_ values were determined as 75 µg/mL for 48 h and 45 µg/mL for 72 and 96 h.

### Gene expression profiling of hepatotoxicity in HepG2 cells treated with EOOO and CV

**Fig. 4:**
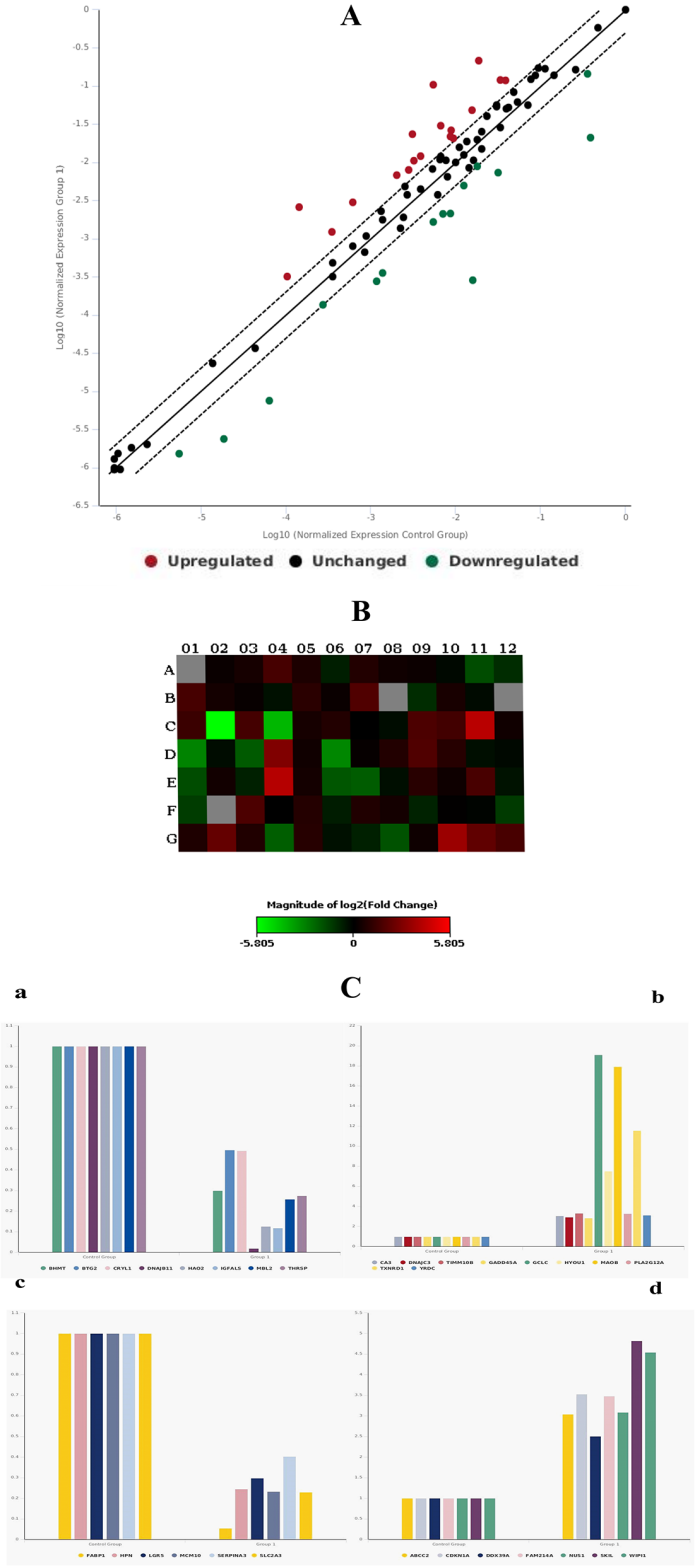
Effects of the EOOO on the gene expression profiles of hepatotoxicity pathway in HepG2 cells. Real-Time PCR based array was performed and the fold changes of the expression of 84 genes were analyzed in the EOOO treated cells comparing with solvent treated control cells. (**A**) A Scatter plot indicates the up-regulated (red dots) and the down-regulated (green dots) genes. (**B**) A heat map indicates the changes of the gene expression on the plate (red for up-regulated and green for down-regulated genes). (**C**) A bar diagram indicates specifically only genes which regulations were changed. The p values are calculated based on a Student’s t-test of the replicate 2^ (- Delta CT) values for each gene in the control group and treatment groups. (**a**) Down-regulated genes directly expressed in hepatotoxicity. (**b**) Up-regulated genes directly expressed in hepatotoxicity. (**c**) Up-regulated genes related to cholestasis, phospholipidosis, necrosis and hepatocarcinogenicity. (**d**) Down-regulated genes related to phospholipidosis, necrosis and hepatocarcinogenicity

**Fig. 5:**
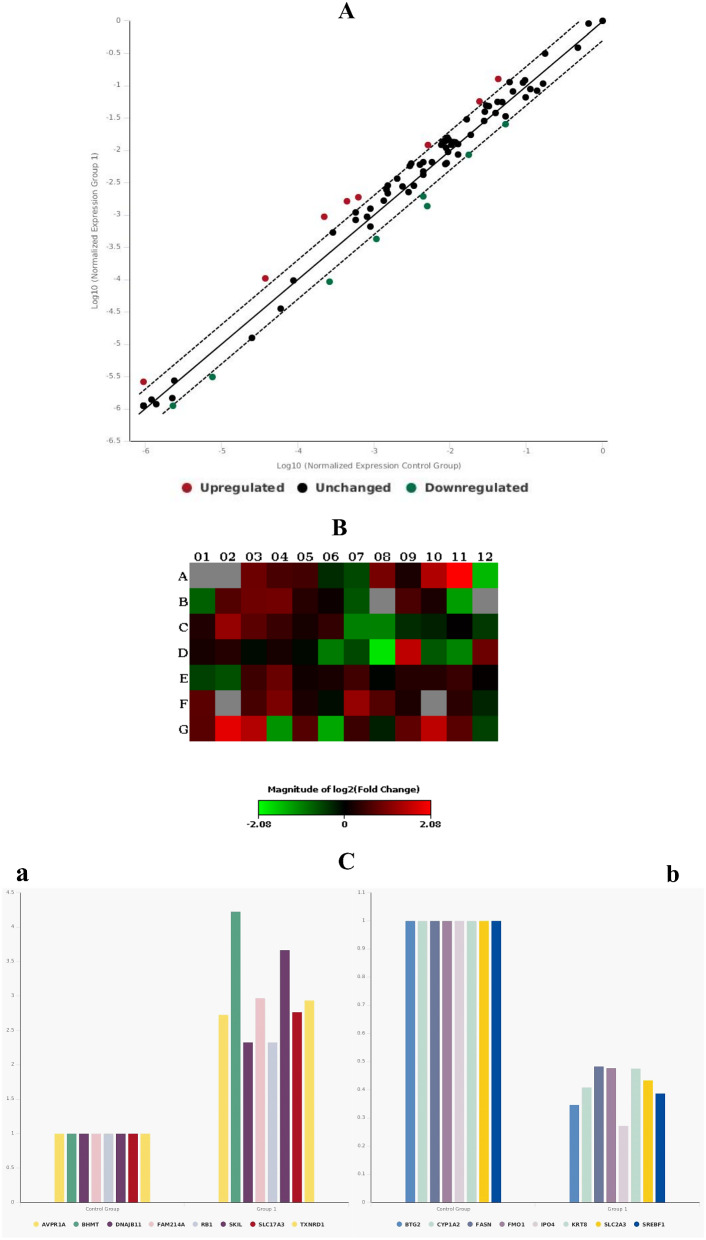
Effects of CV on gene expression profiles of hepatotoxicity pathway. The fold change of the 84 genes in CV compared to control group was evaluated by RT-PCR array analysis. (**A**) The red and green dot stands for up-regulated and down-regulated genes, respectively for 84 genes analyzed (**B**) The red and green color represent for increasing or decreasing genes in the EOOO treatment group against non-treated control group respectively showed in the heat map. (**C**) The bar diagram is the clustering of differential expression genes in hepatotoxicity for up-regulated (**a**) and down-regulated (**b**) genes expressed. The p values are calculated based on a Student’s t-test of the replicate 2^ (- Delta CT) values for each gene in the control group and treatment groups

The differential expression levels of mRNAs of the 84 genes involved in the common hepatotoxicity pathway were assessed in HepG2 cells treated with 0.08 µg/mL EOOO and 45 µg/mL CV for 24 h. The human hepatotoxicity RT^2^ PCR array used profiles the expression of 84 key genes implicated as potential biomarkers of liver toxicity which are related to the common hepatotoxicity pathway including cholestasis, steatosis, nongenotoxic hepatocarcinogeniticity, phospholipidosis and necrosis (Table 2). Also, after an extensive literature search, the genes included in the qRT-PCR array kit were appropriate for the hepatotoxicity pathway model. The PCR array results of 84 mRNAs expressed in EOOO- and CV-treated cells were presented as scatter plots, heat maps, and graphics of fold changes Figs. 4 and 5, respectively. In the analyses, the normalized expression of every gene was compared between two selected groups in duplicates of two separate experiments. A scatter plot indicates the unchanged gene expression in the central line, Up-regulated genes in the upper left as red color, and down-regulated genes in the lower right sections as green (Figs. 4A and 5A). A heat map of each plate provides a visualization of the fold changes in the expression of every gene in the context of the array layout. Red and green colors on the heat maps indicate increasing or decreasing genes, respectively (Figs. 4B and 5B). Regulated genes were specifically expressed as a bar diagram with their counterpart controls (Figs. 4C and 5C).

**Table 2:** Functional grouping of the genes involved in the pathways of hepatotoxicity using human hepatotoxicity RT² profiler PCR array (Qiagen)

Involved mechanism	Genes
Cholestasis	ABCB1, ABCB4, ABCC2, ABCC3, ATP8B1, ICAM1, OSTALPHA (SLC51A), PDYN, RDX.
Steatosis	
*upregulated* *downregulated*	CD36, FASN, LPL, SCD. PPARA, SREBF1.
**Phospholipidosis** *upregulated* *downregulated* *regulated*	ASAH1, FABP1, HPN, LSS, MRPS18B, S100A8, SERPINA3, WIPI1. SLC2A3, TAGLN. ABCB1, FXC1.
**Hepatotoxicity** *upregulated* *downregulated* *regulated*	ALDOA, APEX1, BTG2, CASP3, CCNG1, CRYL1, DDIT4L, DNAJB11, DNAJC3, GADD45A, GCLC, GSR, HMOX1, HYOU1, KRT18, KRT8, NQO1, PLA2G12A, SLC17A3, TXNRD1. YRDC, AVPR1A, BHMT, CA3, CXCL12, CYP1A2, FADS1, FMO1, HAO2, IGFALS, MBL2, RB1, THRSP. ABCB11, FXC1, MAOB, PYGL.
**Nongenotoxic Hepatocarcinogenicity** *upregulated*	ALDOA, APEX1, BTG2, CCNG1, CDKN1A, DDX39, KRT8, KRT18, MRPS18B, TXNRD1.
**Necrosis** *upregulated* *downregulated* *regulated*	CD68, COL4A1, IL6ST, IPO4, MAP3K6, NUS1, OSMR, PSME3, SERPINE1, SKIL, SLC39A6, TMEM2 CDC14B, EMC9, FAM214A, FAM158A, KIAA1370, L2HGDH, LGR5. MCM10, MLXIPL, RHBG. CDKN1A, DDX39.

The scatter plot and the heat map indicate differential regulation of 32 genes (by more than 2-fold) in EOOO-treated cells compared to the solvent-treated control group (Fig. 4A and B). Among them, the expression of 17 genes (ABCC2, CA3, CDKN1A, DDX39A, DNAJC3, TIMM10B, GADD45A, GCLC, HYOU1, FAM214A, MAOB, NUS1, PLA2G12A, SKIL, TXNRD1, WIPI1 and YRDC) were significantly upregulated while the expression of 15 genes (BHMT, BTG2, CRYL1, DNAJB11, FABP1, HAO2, HPN, IGFALS, LGR5, MBL2, MCM10, SLC51A, SERPINA3, SLC2A3, and THRSP) was downregulated (Fig. 4C). The regulation of DNAJB11, FABP1, GCLC, MAOB, TXNRD1, IGFALS, HAO2, and HYOU1 was sharply changed upon treatment with the EOOO of HepG2 cells (Table 3).

**Table 3:** Description of regulated genes of EOOO-treated HepG2 cells and their related pathways

Gene Symbol	Fold Change	Description	RefSeq Accestion		Related Pathways
**Upregulated**					
**GCLC**	**19.09**	Glutamate-cysteine ligase, catalytic subunit	NM_001498	Hepatotoxicity	
**MAOB**	**17.94**	Monoamine oxidase B	NM_000898		
**HYOU1**	**7.52**	Hypoxia up-regulated 1	NM_006389		
**PLA2G12A**	3.27	Phospholipase A2, group XIIA	NM_030821		
**YRDC**	3.12	YrdC domain containing	NM_024640		
**CA3**	3.06	Carbonic anhydrase III, muscle specific	NM_005181		
**DNAJC3**	2.94	DnaJ (Hsp40) homolog, subfamily C, member 3	NM_006260		
**GADD45A**	2.85	Growth arrest and DNA-damage-inducible, alpha	NM_001924		
**TXNRD1**	**11.55**	Thioredoxin reductase 1	NM_003330	Hepatotoxicity and Hepatocarcinogeniticity	
**TIMM10B (FXC1)**	3.32	Translocase of Inner Mitochondrial Membrane 10B	NM_012192	Hepatotoxicity Phospholipidosis	
**CDKN1A**	3.53	Cyclin-dependent kinase inhibitor 1 A (p21, Cip1)	NM_000389	Hepatocarcinogeniticity	
**WIPI1**	4.55	WD repeat domain, phosphoinositide interacting 1	NM_017983	Phospholipidosis	
**SKIL**	4.82	SKI-like oncogene	NM_005414	Necrosis	
**FAM214A** (**KIAA1370)**	3.48	Family with Sequence Similarity 214, Member A	NM_019600		
**NUS1**	3.08	Nuclear undecaprenyl pyrophosphate synthase 1 homolog (S. cerevisiae)	NM_138459		
**DDX39A**	2.51	DEAD (Asp-Glu-Ala-Asp) box polypeptide 39 A	NM_005804	Necrosis Hepatocarcinogeniticity	
**ABCC2**	3.04	ATP-binding cassette, sub-family C (CFTR/MRP), member 2	NM_000392	Cholestasis	
**Downregulated**					
**DNAJB11**	**-55.91**	DnaJ (Hsp40) homolog, subfamily B, member 11	NM_016306		Hepatotoxicity
**HAO2**	**-7.94**	Hydroxyacid oxidase 2 (long chain)	NM_016527		
**MBL2**	-3.88	Mannose-binding lectin (protein C) 2, soluble	NM_000242		
**THRSP**	-3.64	Thyroid hormone responsive	NM_003251		
**BHMT**	-3.34	Betaine–homocysteine S-methyltransferase	NM_001713		
**CRYL1**	-2.03	Crystallin, lambda 1	NM_015974		
**IGFALS**	-1.89	Insulin-like growth factor binding protein, acid labile subunit	NM_004970		
**BTG2**	-2.01	BTG family, member 2	NM_006763		Hepatotoxicity and hepatocarcinogenicity
**FABP1**	-**18.44**	Fatty acid binding protein 1, liver	NM_001443		Phospholipidosis
**SLC2A3**	-4.36	Solute carrier family 2 (facilitated glucose transporter), member 3	NM_006931		
**HPN**	-4.08	Hepsin	NM_002151		
**SERPINA3**	-2.48	Serpin peptidase inhibitor, clade A (alpha-1 antiproteinase, antitrypsin), member 3	NM_001085		
**MCM10**	-4.30	Minichromosome maintenance complex component 10	NM_182751		Necrosis
**LGR5**	-3.35	Leucine-rich repeat containing G protein-coupled receptor 5	NM_003667		
**SLC51A**	-2.55	Solute carrier family 51 alpha subunit	NM_152672		Cholestasis

Description of regulated genes such as gene symbol, RefSeq accession, and their related pathways are given in Table 3. As seen here, among the upregulated genes, eight genes (MAOB, GCLC, HYOU1, PLA2G12A, YRDC, CA3, DNAJC3, and GADD45A) involved in hepatotoxicity pathway, four genes (SKIL, FAM214A, NUS1, and DDX39A) in necrosis, one each in hepatocarcinogenicity (CDKN1A), cholestasis (ABCC2) and phospholipidosis (WIPI1). On the other hand, a gene TIMM10B is involved in both hepatotoxicity and phospholipidosis pathways. TXNRD1 gene is involved in both hepatotoxicity and hepatocarcinogenicity. On the other hand, seven genes (DNAJB11, HAO2, MBL2, THRSP, BHMT, CRYL1, and IGFALS) were downregulated in the hepatotoxicity pathway, two genes (MCM10 and LGR5) in necrosis, four genes in phospholipidosis (FABP1, SLC2A3, HPN, and SERPINA3), one each in hepatocarcinogenicity (BTG2) and cholestasis (SLC51A) pathways.

The scatter plot and the heat map indicate differential regulation of 16 genes (at least 2-fold or more) in CV-treated cells (Fig. 5) compared to the solvent-treated control group (Fig. 5A and B). Among them, the expression of 8 genes (BHMT, SKIL, FAM214A, TXNRD1, SLC17A3, AVPR1A, DNAJB11, and RB1) was significantly upregulated while the expression of 8 genes (IPO4, BTG2, SREBF1, CYP1A2, SLC2A3, KRT8, FMO1, and FASN) was downregulated (Fig. 5C).

The description of regulated genes such as gene symbol, RefSeq accession, and their related pathways are given in Table 4. As seen here, among the upregulated genes, five genes (DNAJB11, AVPR1A, BHMT, SLC17A3, and RB1) are involved in hepatotoxicity pathway and two genes (SKIL and FAM214A) in necrosis. A gene TXNRD1 is involved in both hepatotoxicity and hepatocarcinogenicity. On the other hand, two genes (CYP1A2 and FMO1) were downregulated in hepatotoxicity pathway, two genes (SREBF1 and FASN) in steatosis, one each in necrosis (IPO4) and phospholipidosis (SLC2A3) pathways. On the other hand, two genes (BTG2 and KRT8) are involved in both hepatotoxicity and hepatocarcinogenicity.

**Table 4:** Description of affected genes and the pathways that they are involved in CV-treated HepG2 cells

Gene Symbol	Fold Change	Description	RefSeq Accession	Related Pathways
**Upregulated**				
**BHMT**	4.23	Betaine–homocysteine S-methyltransferase	NM_001713	Hepatotoxicity
**SLC17A3**	2.77	Solute carrier family 17 (sodium phosphate), member 3	NM_006632	
**AVPR1A**	2.73	Arginine vasopressin receptor 1 A	NM_000706	
**DNAJB11**	2.33	DnaJ (Hsp40) homolog, subfamily B, member 11	NM_016306	
**RB1**	2.33	Retinoblastoma 1	NM_000321	
**SKIL**	3.67	SKI-like oncogene	NM_005414	Necrosis
**FAM214A** (**KIAA1370)**	2.97	Family with Sequence Similarity 214, Member A	NM_019600	
**TXNRD1**	2.94	Thioredoxin reductase 1	NM_003330	Hepatotoxicity and Hepatocarcinogenicity
**Downregulated**				
**CYP1A2**	-2.45	Cytochrome P450, family 1, subfamily A, polypeptide 2	NM_000761	Hepatotoxicity
**FMO1**	-2.09	Flavin containing monooxygenase 1	NM_002021	
**BTG2**	-2.88	BTG family, member 2	NM_006763	Hepatotoxicity and Hepatocarcinogenicity
**KRT8**	-2.10	Keratin 8	NM_002273	
**SREBF1**	-2.58	Sterol regulatory element binding transcription factor 1	NM_004176	Steatosis
**FASN**	-2.06	Fatty acid synthase	NM_004104	
**SLC2A3**	-2.31	Solute carrier family 2 (facilitated glucose transporter), member 3	NM_006931	Phospholipidosis
**IPO4**	-3.67	Importin 4	NM_024658	Necrosis

## Discussion

HCC accounts for approximately 90% of all liver cancers, making it one of the most prevalent and rapidly growing types of cancer [45]. Several studies have shown that CV and EOOO exhibit antiproliferative and proapoptotic effects against cancer cells including HepG2 cells and HCC [11, 23, 46, 47]. On the other hand, recently it has been suggested that many commercial herbal products, medicinal plants, or their constituents which have been used for the treatment of diseases including HCC may have potentially hepatotoxic effects [34–36, 38, 39, 48]. Therefore, the possible hepatotoxic effects of compounds from medicinal plants are drawing attention and should be investigated. In this regard, we investigated the anti-proliferative and growth inhibition effects of the EOOO and CV and then, for the first time, demonstrated the mechanisms of hepatotoxicity and hepatocarcinogenicity in HepG2 cells, at the gene expression levels.

In the previous work, we demonstrated the cytotoxic and apoptotic effect of the EOOO and CV performing cell viability and morphological assays on HepG2 cells [40]. Here, in addition to the aforementioned assays, we further showed that both EOOO and CV inhibit DNA synthesis in the cells. Similarly, CV inhibited DNA synthesis in N-ras-transformed mouse myoblasts [22]. Recently, stronger cytotoxic activities of oregano essential oil and CV on HepG2 cells than on health cells have been reported [23, 49]. In the study conducted by Becer et al. [23], a cytotoxic effect was noted at higher doses compared to our findings, alongside the demonstration of an antioxidant effect. This observation might be correlated with the drug resistance of the cells. Furthermore, the pharmacological effects of essential oils are intricately linked to their composition. This finding is consistent with the data reported by Yin et al. who showed that CV (0.05, 0.1, 0.2, 0.4 mmol L-1) inhibited proliferation and induced pro-apoptosis in HepG2 cells. They showed activation of caspase-3, cleavage of PARP, and reduced expression of the Bcl-2 gene [50]. Furthermore, in two separate in vivo studies, CV exhibited hepatoprotective potential in D-galactosamine-induced hepatotoxicity and diethylnitrosamine-induced hepatocellular carcinoma in rats [51, 52]. Despite numerous studies demonstrating the cytotoxic, apoptotic, anticancer, and hepatoprotective effects of EOOO and CV on the liver using various biological systems [11], the molecular mechanism underlying the potential hepatotoxicity of these compounds remains unclear.

In the present study, we investigated and evaluated the expression of a panel of 84 genes involved in the hepatotoxicity pathway, encompassing cholestasis, steatosis, phospholipidosis, hepatotoxicity, nongenotoxic hepatocarcinogenicity, and necrosis mechanisms, in HepG2 cells treated with EOOO or CV. According to RT-PCR array results, treatment of the cells with both EOOO and CV resulted in the amelioration of many hepatotoxicity-related gene expressions. In EOOO-treated cells, 15 key genes were upregulated while 17 others were downregulated. In CV-treated cells, 8 genes were upregulated while 8 others were downregulated. These variances in the effects of EOOO and CV are anticipated due to the diverse compounds present in essential oils, which may exhibit synergistic effects.

The expression of 14 genes (GCLC, MAOB, HYOU1, CA3, PLA2G12A, YRDC, DNAJC3, GADD45A, TIMM10B, CDKN1A, WIPI1, NUS1, DDX39A, and ABCC2) was exclusively upregulated following EOOO treatment of the cells. Among these genes, the upregulation of GCLC, MAOB, HYOU1, CA3, PLA2G12A, YRDC, DNAJC3, and GADD45A is associated with hepatotoxicity. GCLC encodes an enzyme responsible for glutathione (GSH) synthesis, playing a crucial role in the anti-oxidation mechanism, as well as in inflammatory and immune response in the liver. Its downregulated expression is used as a marker for HCC [53]. GCLC is transcriptionally upregulated by nuclear factor-erythroid factor 2-related factor 2 (Nrf2) which offers protection against liver injury. Additionally, phenolic compounds have been shown to influence the cellular redox mechanism [54]. Linalool, a component of EOOO, has been shown to activate the Nrf2 signaling pathway during lung inflammation and increase the level of ROS while simultaneously decreasing the level of GSH in HepG2 cells [55, 56]. Additionally, CV has been found to induce ROS generation by decreasing glutathione levels through its pro-oxidant capacity in human gastric adenocarcinoma and fibroblast cells [57]. The monoamine oxidase B (MAOB) and hypoxia upregulated 1 (HYOU1) have been reported to be upregulated in hepatotoxicity as well as in various cancers [14, 58]. CA3 plays a role as an antioxidant in skeletal muscle injury and protects cells from hydrogen peroxide-induced apoptosis. However, the expression of CA3 was found to be reduced in HCC [59]. PLA2G12A encodes phospholipase A2 which is responsible for liberating arachidonic acid from phospholipids. It is highly expressed during inflammation and toxicity [60]. YRDC, on the other hand, is expressed ubiquitously in several human tissues including the liver and pancreas and its expression has been suggested as a biomarker of HCC [61]. DNAJC3 encodes another heat shock protein (hsp40) and is upregulated after exposure to cadmium and coumarin in rat liver cells in vivo and in vitro [62]. GADD45A, which encodes the nuclear protein (growth arrest and DNA-damage-inducible protein 45 alpha) plays a crucial role in maintaining genomic stability, DNA demethylation, DNA repair, and suppression of cell growth. It has been found to be upregulated in human hepatocytes treated with anti-inflammatory drugs, as a target gene of p53 [63], as well as in thioacetamide-induced hepatotoxicity in rats [64, 65]. TIMM10B, also known as FXC1, encodes an enzyme that is a subunit of complexes in the mitochondrial intermembrane translocase. It is regulated in both hepatotoxicity and phospholipidosis. Recently, a high level of TIMM10B expression was found during diclofenac-induced hepatotoxicity [66]. TIMM10B, also known as FXC1, encodes an enzyme that serves as a subunit of complexes in the mitochondrial intermembrane translocase. It is regulated in both hepatotoxicity and phospholipidosis. Recently, a high level of TIMM10B expression was found during diclofenac-induced hepatotoxicity [66]. CDKN1A encodes a potent cyclin-dependent kinase inhibitor, p21/WAF1, which is regulated by the tumor suppressor p53. Its down-regulation is associated with tumor progression in human HCC [67]. WIPI1 gene encodes a protein with several biochemical functions, including phosphatidylinositol-3,5-phosphate binding, indicating its involvement in phospholipidosis. One of its significant roles is to regulate autophagosome formation, which is significantly associated with cancer. WIPI1 gene was also found to be upregulated in HCC patients [68, 69]. NUS1 gene encodes a dehydrodolichyl diphosphate synthase subunit which is essential for dolichol synthesis and protein glycosylation. Its expression is associated with necrosis and the poor prognosis of human hepatocellular carcinoma patients [70]. DDX39 was initially identified as a novel growth-associated RNA helicase and is found to be overexpressed in lung squamous cell carcinoma and telomerase-positive human cancer cells [71]. ABCC2 gene has been implicated in drug and estrogen-induced cholestasis [72]. Furthermore, EOOO treatment may induce activation of certain genes responsible for primarily hepatotoxicity and hepatocarcinogenicity in HepG2 cells.

The expression of 11 genes (HAO2, MBL2, THRSP, CRYL1, IGFALS, FABP1, HPN, SERPINA3, LGR5, MCM10, and SLC51A) was decreased exclusively following EOOO treatment of cells. The downregulation of HAO2, MBL2, THRSP, and IGFALS genes, as observed in our study, has been associated with hepatotoxicity. Additionally, the reduced expression of HAO2 and IGFALS has been suggested as a potential prognostic and diagnostic marker for hepatocellular carcinoma (HCC) [73, 74]. MBL2 encodes an important protein that has a protective role in the first stages of hepatitis virus infection and its expression was downregulated in HCC [75, 76]. THRSP encodes a nuclear protein involved in regulating of genes required for fatty acid synthesis. Its expression has been found to be downregulated in human breast cancer and liposarcoma cells [77]. Overexpression of THRSP indicates triglyceride accumulation with lipogenesis [78]. CRYL1 encodes an enzyme, crystallin lambda 1, which plays a role in urinate cycle. Reduced expression of CRYL1 has been observed in HCC [79]. Additionally, the downregulation of FABP1, HPN, and SERPINA3 genes is related to phospholipidosis. We observed a significant decrease in the expression of FABP1 following EOOO treatment of the cells, indicating the potent hepatoprotective effect of EOOO. FABP1 serves as a potential regulator of lipid metabolism and is prominently expressed in the liver. The reduction in its expression further suggests its protective role against liver injury [80]. HPN, hepsin, has frequently over-expressed in many cancers but is decreased in HCC cells [81, 82]. SERPINA3 has been found upregulated in phospholipidosis. Additionally, two other genes downregulated by EOOO, MCM10 and LGR5 are associated with necrosis. MCM10 is involved in DNA replication and is highly expressed in HCC tumors [83]. The overexpression of LGR5 has been shown to promote HCC metastasis [84]. These results suggest the potential anti-cancer activity of EOOO in HCC. SLC51A also known as OSTalpha is widely expressed in the human small intestine and liver and is a major transporter of bile acids associated with cholestasis. Studies have shown that SLC51A-deficient mice display significantly decreased levels of bile acids [85, 86].

Out of the 37 hepatotoxicity-related genes analyzed, the differential regulation of fourteen genes (nine upregulated and five downregulated) suggests a potential hepatotoxic effect of EOOO. Conversely, the regulation of three genes (CA3, DNAJB11, and BHMT) indicates the inhibition of hepatotoxicity. Two genes (TXNRD1 and CDKN1A) out of 10 nongenotoxic hepatocarcinogenic genes may suggest the effect of EOOO on carcinogenicity whereas regulation of a gene BTG2 indicates the inhibition of carcinogenicity. Among the 12 phospholipidosis-related genes analyzed, the differential regulation of three genes (two upregulated and one downregulated) may indicate the effect of EOOO on phospholipidosis, while the regulation of three other genes suggests the inhibition of phospholipidosis. Additionally, the EOOO may play a role in the regulation of lipid metabolism and glutathione synthesis. Regarding necrosis, five genes out of 22 necrosis genes may suggest the necrotic effect of EOOO while the downregulation of the FAM214A genes indicates the anti-necrotic effects. Furthermore, the expression only 2 genes out of 9 cholestasis genes was affected by EOOO, with one being upregulated and the other downregulated, indicating a relatively weak influence of EOOO on cholestasis.

In CV-treated cells, the expression of 3 different genes (AVRP1A, SLC17A3, and RB1) involved in hepatotoxicity was upregulated while 6 different genes (CYP1A2, FMO1, KRT8, FASN, SREBF1, and IPO4) were downregulated. AVPR1A encodes a receptor that mediates cell contraction and proliferation, platelet aggregation, and glycogenosis, and its expression was associated with drug use disorders [87]. SLC17A3 (known as hNPT4) acts as a voltage-driven transporter for drugs and urate [88]. RB1 is a tumor suppressor gene and encodes a protein that is a negative regulator of the cell cycle [89]. The downregulation of CYP1A2 and FMO1 is associated with hepatotoxicity. CYP1A2 encodes a member of the cytochrome P450 family, which plays a crucial role in drug metabolism and lipid synthesis in the liver [90, 91]. FMO1 protein is a member of flavin-containing monooxygenases and play a significant role in the metabolism of small molecule pharmaceuticals [82]. KRT8 encodes keratin 8 and its upregulation is associated with both hepatotoxicity and nongenotoxic hepatocarcinogenicity. KRT8 was highly expressed in HCC [92], on the other side absence of its expression led to the development of mild liver injury [93]. The downregulation of FASN and SREBF1 is involved in hepatic steatosis. FASN encodes a key enzyme for catalyzing endogenous fatty acid synthesis, high-level expression influences the migration, and invasion of HCC cells. SREBF1 protein is a transcription factor and induces the activation of genes involved in sterol biosynthesis including the low-density lipoprotein receptor gene [82, 94]. IPO4 encodes importin 4 and its reduced expression is implicated with necrosis and Vitamin D deficiency in chronic liver disease [95, 96]. CV treatment may play a role in liver injury through the mechanism involving Vitamin D.

Recent research indicated has highlighted the antioxidant and hepatoprotective properties of CV in galactosamine-hepatotoxic rats [51]. Additionally, HepG2 cells serve as a crucial hepatocyte model for drug metabolism and hepatotoxicity studies owing to their manifestation of fundamental characteristics of hepatocytes [97, 98]. An experiment showcased that thymol, constituting 7.6% of the EOOO, along with CV, protect HepG2 cells against acetaminophen-induced toxicity by mitigating oxidative stress, and enhancing antioxidant defense mechanisms [99]. Furthermore, a recent study delineated the hepatoprotective effect of CV against carbon tetrachloride-induced liver toxicity in albino rats evidenced by the reduction in serum ALT and AST levels, possibly mediated through its antioxidant effect, as validated by the elevation in liver GSH levels. Additional studies concerning hepatotoxicity have reported the augmentation of CV concentration of liver regeneration and its protective effects. Moreover, another in vivo study also showcased that CV play a protective role following liver ischemia/reperfusion [100, 101].

Taken together, our results indicate that CV exerted a hepatotoxic effect by regulating five genes out of 37 hepatotoxicity gene panels while simultaneously inhibiting hepatotoxicity by regulating five other genes. Additionally, CV affected non-genotoxic hepatocarcinogenicity by regulating the expression of TXNRD1 and BTG2 out of 10 genes studied. However, it inhibited carcinogenicity by regulating the expression of numerous genes. Two genes out of 22 necrosis-related genes suggest the anti-necrotic effect of CV. Conversely, there is no observed of CV on cholestasis. The upregulation of SLC2A3, out of the 12 phospholipidosis-related genes, highlights the significant impact of CV on phospholipidosis. These findings may support the claim of anti-obesity activity associated with CV. Moreover, the upregulation of SREBF1, out of 10 steatosis-related genes suggests a steatotic effect of CV. However, the regulation of a gene FASN gene indicates an inhibitory effect on steatosis. The downregulation of FASN by CV is an important indicator for its possible anti-cancer activities particularly given its high expression in HCC.

In both EOOO and CV-treated cells, three genes, TXNRD1, FAM214A, and SKIL, exhibited upregulated, while two genes, BTG2 and SLC2A3 were downregulated. TXNRD1 gene is pivotal in maintaining cellular redox balance, which can be disrupted by the generation of reactive oxygen species (ROS) generation and oxidative damage. Furthermore, TXNRD1 is also a biomarker for liver injury and has been documented to be overexpressed in HCC [102, 103]. SKIL gene is a proto-oncogene belonging to the Ski protein family. It was reported that the downregulation of SKIL promotes cell growth inhibition and apoptosis in HCC cells [104]. FAM214A is a family with sequence similarity 214 member A and was found to be upregulated in hepatotoxicity in rats after treatment with *Dioscorea hispida* extract [105]. An association between FAM214A and non-genotoxic hepatocarcinogenicity in mice has been reported [106]. BTG2 encodes anti-proliferation factor 2 and is upregulated during hepatotoxicity and found to be downregulated in HCC [107]. SLC2A3 has been associated with the Nrf2 pathway and found to be upregulated during phospholipidosis [82, 108]. On the other hand, in EOOO-treated cells, two genes related to hepatotoxicity, BHMT, and DNAJB11 were downregulated, whereas they were upregulated in CV-treated cells. BHMT serves as a prognostic biomarker for HCC and liver injury, and its expression was found to be downregulated in HCC patients [109]. DNAJB11 encodes a heat shock protein, and studies have shown that silencing of the gene reduces the proliferation of human HCC cells [110]. Additionally, upregulation of DNAJB11 associated with a protective role against cellular stresses, including oxidative stress [111].

Our findings suggest that the EOOO and CV did not suppress the high expression of SKIL and TXNRD1 genes associated with HCC. However, they did ameliorate the expression of many genes related to hepatotoxicity. Moreover, the expression of numerous genes in response to hepatocarcinogenicity was suppressed by the EOOO and CV. Nevertheless, it’s important to note that the effects of EOOO and CV are differed. These results imply that other components of the EOOO, such as thymol and linalool, may also possess anti-cancer or different activity [14, 15, 58]. Therefore, other components of EOOO may induce the expression of different genes involved in hepatotoxicity pathways. Additionally, the EOOO did not affect related to steatosis. It is plausible that CV and other components of EOOO may synergistically ameliorate gene expression in response to steatosis. Kim et al. reported that CV may have a chemopreventive role in hepatic steatosis, which can lead to steatohepatitis, effectively serving as a risk factor for HCC. [112]. Jayakumar et al. also investigated the chemopreventive effect of CV on diethylnitrosamine-induced liver cancer in albino rats [52]. It was found that CV has potential free radical scavenging and antioxidant effects and further enhances the defense mechanism. According to our results, the EOOO may play an important role in modulating oxidative stress genes through its impact on the redox mechanism.

## Conclusion

In conclusion, the present study demonstrated the anti-proliferative and DNA-synthesis inhibitory effects of the essential oil from *Origanum onites* L. and its component CV on a hepatocellular carcinoma cell line, HepG2. Consequently, both the EOOO and mainly CV show promise as potential to be anticancer agents. Intriguingly, our hepatotoxicity array results indicated that neither EOOO nor CV induced the expression of many hepatotoxicity-related genes, suggesting that they might not exert hepatotoxic effects on HCC cells. Moreover, the downregulation of numerous carcinogenicity-related genes in both EOOO and CV-treated cells suggests their potential anticancer effects. Furthermore, the amelioration of several hepatotoxicity-related genes by both EOOO and notably by CV treatments strongly suggests their hepatoprotective effects. Despite numerous studies documenting the anti-hepatotoxic effects of CV, the precise molecular mechanisms underlying its action remain elusive. Hence, this study represents the first attempt to investigate the effects of EOOO and CV on the expression of genes implicated in the hepatotoxicity model. Consequently, we suggest that both EOOO and CV, as herbal drugs, or products, hold promise as non-hepatotoxic anticancer agents. To gain a deeper understanding of the exact non-hepatotoxic mechanisms of EOOO and CV, more sophisticated studies utilizing additional methods such as GSH analysis, cytokine array, ROS, MDA and MMP detection, as well as single-cell RNA sequencing, are warranted to decipher hepatotoxicity.

## Data Availability

All data generated or analyzed during this study are included in this published article.

## Abbreviations

AP-1: Activator Protein-1
BrdU: Bromodeoxyuridine
COX-2: Cyclooxygenase-2
CV: Carvacrol
DMEM: Dulbecco Modified Eagle Medium
EOOO: Essential oil of Origanum onites
ERK1/2: Extracellular signal-regulated kinase 1/2
FBS: Fetal bovine serum
FOXP3: Forkhead box protein P3
GC: Gas Chromatography
GC-MS: Gas Chromatography-Mass Spectrometry
GSH: Glutathione
HCC: Hepatocellular carcinoma
HYOU1: Hypoxia up-regulated 1
IFN-γ: Interferon gamma
IL: Interleukin
JNK: c-Jun N-terminal kinases
MAPK: Mitogen-activated protein kinase
MAOB: Monoamine oxidase B
Nrf2: Nuclear factor-erythroid factor 2-related factor 2
PCR: Polymerase chain reaction
PLC-γ: Phospholipase C gamma
PPAR-γ: Peroxisome proliferator- activated receptor gamma
TB: Trypan blue
RRI: Relative retention index
RT: Real time
RT2: Real-time, reverse-transcription
TGF-β: Transforming growth factor beta
TLR: Toll like receptor
